# Supporting and retaining Village Health Teams: an assessment of a community health worker program in two Ugandan districts

**DOI:** 10.1186/s12939-017-0619-6

**Published:** 2017-07-20

**Authors:** Daniel C. Mays, Edward J. O’Neil, Edison A. Mworozi, Benjamin J. Lough, Zachary J. Tabb, Ashlyn E. Whitlock, Edward M. Mutimba, Zohray M. Talib

**Affiliations:** 1 George Washington University School of Medicine & Health Sciences, 2300 Eye St. NW, Washington D.C., 20037 USA; 2 Omni Med, 81 Wyman St #1, Waban, MA 02468 USA; 3 Department of Paediatrics and Child Health, Makerere University College of Health Sciences, P.O. Box 7072, Kampala, Uganda; 4 University of Illinois at Urbana-Champaign, School of Social Work, 1010 W. Nevada Street, Urbana, IL 61801 USA; 5 Warren Alpert Medical School of Brown University, 222 Richmond St, Providence, RI 02903 USA; 6 Omni Med Uganda, Kisoga Town, Mukono District Uganda

**Keywords:** Community health workers, Village health teams, Community health volunteers, Uganda, Sustainability, Retention, Support, Attrition, Health workforce

## Abstract

**Background:**

Uganda’s national community health worker program involves volunteer Village Health Teams (VHTs) delivering basic health services and education. Evidence demonstrates their positive impact on health outcomes, particularly for Ugandans who would otherwise lack access to health services. Despite their impact, VHTs are not optimally supported and attrition is a growing problem. In this study, we examined the support needs and existing challenges of VHTs in two Ugandan districts and evaluated specific factors associated with long-term retention. We report on findings from a standardized survey of VHTs and exploratory interviews with key stakeholders and draw conclusions that inform efforts to strengthen and sustain community health care delivery in Uganda.

**Methods:**

A mixed-methods approach was employed through a survey of 134 individual VHT members and semi-structured interviews with six key stakeholders. Descriptive and bivariate regression analysis of quantitative survey data was performed along with thematic analysis of qualitative data from surveys and interviews. In the regression analysis, the dependent variable is 10-year anticipated longevity among VHTs, which asked respondents if they anticipate continuing to volunteer as VHTs for at least 10 more years if their current situation remains unchanged.

**Results:**

VHTs desire additional support primarily in the forms of money (e.g. transportation allowance) and material supplies (e.g. rubber boots). VHTs commonly report difficult working conditions and describe a lack of respect from their communities and other health workers. If their current situation remains unchanged, 57% of VHTs anticipate remaining in their posts for at least 10 years. Anticipated 10-year longevity was positively associated with stronger partnerships with local health center staff and greater ease in home visiting.

**Conclusions:**

Supporting and retaining Uganda’s VHTs would be enhanced by building stronger partnerships between VHTs and other health workers and regularly providing supplies and transportation allowances. Pursuing such measures would likely improve equity in access to healthcare for all Ugandans.

**Electronic supplementary material:**

The online version of this article (doi:10.1186/s12939-017-0619-6) contains supplementary material, which is available to authorized users.

## Background

Uganda, an East African nation home to nearly 40 million people, is one of many countries facing a critical shortage of health workers and a heavy burden of morbidity and mortality [1, 2]. Low- and middle-income countries (LMICs) have responded to these shortages by utilizing community health workers, a diverse group of health workers equipped to provide basic health interventions in local neighborhoods and communities [3]. Uganda founded the Village Health Team community health worker program in 2001 in an effort to bridge the gap created by the health worker shortage and improve equity in access to health services [4]. Within Uganda’s tiered national health system, the Village Health Team holds the position of Health Centre I, followed by Health Centres II-IV, which are local clinics, each with sequentially higher levels of capacity and larger catchment areas [5]. Similar to other types of community health workers (CHWs) throughout the Global South, Village Health Team members (VHTs) “mobilize communities for health programs and strengthen the delivery of health services at household level” [3, 4]. After basic training, each VHT member is assigned a cohort of households in his or her home community. VHT responsibilities include recording demographic and health data, educating on health and hygiene topics, mobilizing families to engage in health programs such as vaccination campaigns, monitoring for illness, making referrals, and providing post-discharge follow up [4]. Since the program’s inception in 2001, over 179,000 individual VHTs have been trained and, as of 2015, VHTs operate in all of Uganda’s 112 districts [4, 5]. Distinct among some CHW programs, VHTs are unpaid volunteers who typically maintain other daily occupations such as farming or shopkeeping [3, 5].

Globally, CHWs in various forms have made powerful contributions to reducing morbidity and mortality, particularly in aspects of maternal and child health and infectious disease management in low-income countries [3, 6]. In Uganda, VHTs have demonstrably increased access to health services and improved health outcomes. Their contributions have been most prominent in reducing morbidity and mortality in children under five as well as managing HIV, TB, and malaria [4, 7–9].

While maintaining measurable health impact, the VHT program faces significant challenges. The program is designed to rely heavily on volunteerism but funding is required for VHT trainings, supplies such as mosquito nets and antimalarial drugs, and stipends for transportation costs. A 2015 Ministry of Health (MOH) assessment concluded, “the VHT strategy has been implemented to varying levels across the districts. Funding of the programme by the government has been gradually reducing since its inception, leaving the IPs [implementing partners] to fund most of the activities” [4]. As financing has fallen primarily to disconnected IPs, there are notable inconsistencies in the allocation of transportation allowances or drugs to distribute. Regarding attrition, the MOH assessment estimates that, among all VHTs trained since 2001, approximately 30% have now abandoned the position. The MOH recommends stronger government commitment to financing the VHT program and a “standardized and harmonized regular and equitable financial form of motivation” for VHTs along with equitable provision of other forms of motivation, such as uniforms, bicycles, and health and hygiene supplies.

Globally, there has been extensive discussion on whether CHWs should be paid as a general rule. While monetary incentives can increase CHW retention, they can also create pitfalls related to sustainability, equity, and relationships with the community [10–13]. Non-monetary incentives are essential to the success of a CHW program, and ultimately, the effectiveness of CHW programs relies upon the relationships between CHWs and the community they serve [14]. The WHO concluded that “appropriate incentives” are critical for program effectiveness, regardless of where health workers fall on the spectrum between volunteers and paid employees [15]. The WHO Workforce 2030 global strategy emphasizes the need to improve working environments and incentives for community-based health workers and calls for context-specific data on the practice environment of community health workers [16].

In Uganda, VHTs are notably motivated by social prestige and responsibility, contributing to improvements in health, receiving education, a hope for career advancement, and hope for paid opportunities or allowances in the future. Common de-motivating challenges involve transportation and health commodity stock-outs [17–19]. Desire for compensation among VHTs is complex, including offsetting time and transportation costs, providing for their families, and feeling appreciated [17]. Remuneration is not a major motivator for most VHTs [20]. Singh and colleagues found that, through partnership with the community and supportive supervision from other health workers, financial motivators such as transport allowances were less important compared to gaining and sharing knowledge, building community relationships, and taking action for better health [21]. A retrospective review found that VHTs can be retained for approximately 5 years without being paid [19]. Further study is needed on the factors associated with VHT retention [4, 19].

The objective of this study is to better understand the support needs and existing challenges of VHTs and identify factors associated with long-term retention. We examined these questions through a mixed-methods approach, using a standardized survey of VHTs in two Ugandan districts and exploratory interviews with key stakeholders. In answering these questions, we aim to inform efforts to strengthen equitable community health care delivery in Uganda.

## Methods

### Study aim and design

This study assessed the opinions and experiences of Village Health Team members (VHTs) and key stakeholders regarding support needs, existing challenges, and factors associated with long-term retention. Between March and May of 2015, this mixed-method study distributed a standardized survey to VHT respondents across two districts in Uganda and conducted six semi-structured key informant interviews (KIIs). The survey was developed by the authors according to issues specific to the VHT program as well as drawing on data from other contexts [3]. The survey consisted of 29 questions, including categorical responses, 1–5 Likert scale rating items, and open-ended free responses (Additional file 1). The survey instrument was translated into the local language, Luganda, and back-translated to ensure accuracy. The survey included VHT demographics, responsibilities, challenges, the nature of existing support, unfulfilled support needs, and experiences engaging at local health centers. With the exception of parish of residence, gender, and age range, no identifying information was recorded. The survey was piloted among a purposive sample of VHTs in the Mukono district in a parish that was purposely excluded from the pool of parishes randomly selected for the study.

### Sampling and recruitment

The Mukono and Wakiso districts, both located in the central region near the capital, were selected among Uganda’s 112 districts based on geographical convenience. Both districts each have approximately 2500 active VHTs, which is consistent with the distribution of VHTs throughout most of the country [4]. A total of 18 parishes were randomly selected from a list of all parishes in the Mukono and Wakiso districts. A list of all VHTs in each parish was obtained from local government officials and NGO program managers. Each list of VHTs from the selected parishes was randomized. Recruitment phone calls were made to each VHT, starting at the top of each randomized parish list until a majority of the VHTs in each parish were successfully recruited. Inclusion criteria were status as an active VHT for more than 6 months. During the recruitment process, VHTs were notified that, upon survey completion, they would receive a transportation stipend of 3000 Ugandan shillings (USD 1.0) [22].

For KIIs, a convenience sample was taken of representative non-governmental organization (NGO) program managers and civil servants from the local- and national-levels of Ugandan government. All of these informants were recruited through email invitations and, after accepting, were interviewed in their offices or over the phone. Informants were neither offered incentives nor provided compensation for participating in the interviews.

### Data collection

Prior to commencing with the interview or survey, subjects provided written informed consent. One survey was administered to each VHT respondent. Surveys were administered by the PI and a data collection assistant who was fluent in the local language, Luganda. In each parish, surveys were administered to all respondents at a centrally convenient meeting place, typically a health center.

The KIIs were semi-structured and exploratory, focusing on the history of the VHT program, its successes and challenges, and opportunities to strengthen the VHT program. The guide for these interviews was designed by the study authors based on existing literature and experience in the field [3–5]. KIIs were held in English as all informants were fluent. Interviews occurred in person or over the phone and were audio recorded with permission.

### Data analysis

Quantitative survey data was analyzed using SPSS software. The primary dependent variable, 10-year anticipated longevity among VHTs, asked if respondents anticipated continuing to volunteer as VHTs for at least 10 more years if their current support remains unchanged. Response options included “yes,” “no,” and “I do not know.” Similar measures have been used in other surveys assessing health worker retention [23, 24]. For analysis, this outcome was recoded to a binary response by coding “no” or “I do not know” = 0 and “yes” = 1. Although VHTs cannot be expected to precisely predict their longevity, a lengthy 10-year prediction was chosen to more rigorously assesses the extent to which VHTs feel equipped and satisfied in their work.

A bivariate regression analysis, t-tests, and chi-square tests were performed, evaluating for associations between categorical responses, e.g. likert item ratings, and the primary dependent variable, i.e. anticipated longevity. A multivariate analysis yielded no significant results, likely due to interrelatedness between multiple independent variables leading to auto correlation when entered into the same model. District was found to be a confounding variable in associations between categorical ratings and anticipated longevity and results were subsequently stratified by district.

Thematic analysis of qualitative data from free response VHT survey questions and transcripts of KIIs was performed using Nvivo 10™ software, employing an applied methodology with a phenomenological framework [25, 26]. This data was triangulated between at least two different coders. Conflicted coding was resolved through dialogue between the coders to reach a consensus [27]. This qualitative interview and free response data was mixed with quantitative survey data using a convergent parallel design whereby qualitative and quantitative data are gathered concurrently and contribute equally to the interpretation and conclusions of the study [28].

### Data management

Each VHT respondent hand-wrote his or her responses onto a paper survey and these were subsequently translated where appropriate and transcribed into a secure Microsoft Excel file. Once transcribed, paper surveys were securely stored. KII audio recordings were transcribed on secure Microsoft Word documents and then deleted. All study documents were saved on a password-protected computer in the possession of the PI. All study data was password-protected and shared securely between study authors.

### Ethical considerations

Ethics approval for the study design, recruitment, and methods was obtained from The George Washington University Institutional Review Board (IRB) and the Uganda National Council for Science and Technology Ethics Committee. During recruitment, it was clarified to each person that there was no risk in declining to participate and no advantage in agreeing to participate apart from the aforementioned transportation stipend for VHT participants. One of the key ethical considerations in this study was to obtain rich data on the experience of VHTs and stakeholders without compromising privacy. Thus, all participants were fully informed of the purpose of the study and the intention for publication. The study was designed such that responses remained anonymous, and respondents were reminded of this aspect throughout the data collection.

## Results

### Descriptive analysis of VHT survey

One hundred thirty four VHTs completed the survey, representing approximately 60% of the VHTs in each of the 18 randomly selected parishes. Descriptive statistics of VHT respondents are presented in Table 1.

**Table 1:** Descriptive statistics of VHT respondents

VHT Demographics	*N*	*(%)*	
VHT Age			
18–29	12	9	
30–39	33	25	
40–49	43	32	
50–59	35	26	
≥ 60	10	8	
VHT Gender			
Female	85	63	
Male	49	37	
VHT District			
Wakiso	46	34	
Mukono	88	66	
VHT Status	*Mean*	*Median*	*IQR*
Years as a VHT	4.22	4	2.5
Households monitoring^a^	94.87	40	53
Typical hours per week on VHT activities^b^	10.41	6.75	8.5

*IQR* inter-quartile range

^a^Each VHT is assigned a cohort of households to monitor for illnesses, provide necessary referrals, and advise on issues of household sanitation and hygiene

^b^As VHTs have other occupations in addition to this volunteer work, respondents were asked to estimate the number of hours spent on VHT activities in a typical week. This is an unverified estimate

The most common sources of livelihood for VHT subjects were one or more of the following: farming or fishing for subsistence (64%), farming or fishing for sale of produce (52%), and running a small business (22%). The most commonly performed VHT activities were mobilizing the community to utilize health services, VHT meetings, distributing health commodities, education, and home visiting. Several motivations for volunteering as a VHT were predominantly rated as “very important:” improving health and saving lives (88%), helping family and friends (87%), and learning useful skills (85%). Less than 40% of respondents rated financial gain and career development as “very important.”

Eighty-three percent of VHT respondents reported referring sick people to the health center, and 55% reported regularly volunteering at the health center. Among these, the most commonly reported task was teaching and counseling patients. Other reported tasks were cleaning the health center, taking measurements, and registering patients.

### Existing support and additional support needs for VHTs

VHTs receive support in various forms and from various sources. Table 2 shows the mean ratings of existing support.

**Table 2:** Ratings of existing support

*Rate the support you currently receive from the following groups*	*Mean rating*	*Standard deviation*	*“Good support” or “the best support” (%)*	*“No support” or “a little support” (%)*
1 = no support; 2 = a little support; 3 = in the middle; 4 = good support; 5 = the best support				
The local health center	3.10	1.49	43.9	37.7
The District Health Office/Ministry of Health	3.03	1.41	42.3	41.5
My family and friends	2.72	1.49	38.0	53.0
My local community	2.71	1.35	32.6	48.5
*Rate the following based on how much support you received in the last year* 1 = none; 2 = a little; 3 = in the middle; 4 = almost enough; 5 = enough	*Mean rating*	*Standard deviation*	*“Almost enough” or “enough” (%)*	*“None” or* *“a little”* *(%)*
Supplies to distribute, e.g. deworming tablets or ITNs	3.78	1.2	70.2	19.8
Partnership with staff at the local health center	3.51	1.30	57.8	28.9
Learning useful skills	3.21	1.48	53.4	38.2
Meeting with other VHTs	3.08	1.32	43.9	41.7
Supervision	3.01	1.42	46.6	42.7
Receiving respect and appreciation	2.84	1.44	36.8	46.1
Transportation tools, such as bicycles or boots	1.52	1.10	10.0	88.5

Respondents also noted other organizations that provide support, such as UNICEF and TASO (The AIDS Support Organization). Notable among these were Omni Med (*n* = 15) and Malaria Consortium (*n* = 8), both responsible for training and managing VHTs in the Mukono and Wakiso districts, respectively. Malaria Consortium is a London-based NGO operating in Wakiso and 16 other districts in Uganda as well as several other countries. Its VHT programs typically have a more limited timeframe and are supported by bilateral and multilateral aid organizations, e.g. UNICEF. Omni Med is an NGO incorporated in Uganda and Boston whose operational scope is limited to the Mukono district. It is funded by individual donors in the U.S. and its timeframe is ongoing.

When asked about additional support needs for better performing VHT work, 66% (*n* = 88) discussed additional monetary support and 60% (*n* = 80) discussed material supplies. Many respondents discussed multiple items in both categories. Among the 88 respondents desiring monetary support, most discussed a transportation allowance (47%), while others discussed a regular salary (27%) or monetary token of appreciation (18%). Among the 80 respondents desiring additional material supplies, the most commonly mentioned items were rubber boots (“gumboots”) (36%), bicycles (35%), umbrellas (25%), treatments to distribute (24%), and uniforms (21%).

- “When you get that little money, sometimes it helps you and motivates you to continue moving in the village even if you are abused or minimized.” (Participant 001, VHT member)
- “...[G]umboots because we are tired of stepping in dirty places.” (Participant 091, VHT member)
- “...[G]iving us medicine in our areas because people come and ask for drugs from us VHTs.” (Participant 007, VHT member)

### Anticipated longevity and bivariate analyses

Fifty-seven percent of respondents reported positive 10-year anticipated longevity (AL), i.e. prediction of continued VHT volunteering for at least 10 years if there is no change in existing support. The other 43% reported negative 10-year AL, i.e. either prediction of discontinuing volunteering within 10 years or uncertainty of continued volunteering. Among this latter group, 62% (27% of study population) reported that they would be able to continue for at least 10 years if they received a monthly payment. Desired amounts ranged from 10,000 to 100,000 Ugandan shillings (USD 3 – USD 30) [22]. Others with negative 10-year AL reported that a transportation allowance or other materials such as rubber boots or bicycles would convert their prediction to positive 10-year AL.

Tables 3 and 4 summarize the bivariate analysis of survey variables, including those from Table 2, and their associations with 10-year AL. The variables shown include those originally hypothesized to be positively associated with positive 10-year AL.

**Table 3:** Bivariate analysis of associations between VHT factors and 10-year anticipated longevity: categorical variables

*Categorical variable*	*Proportion with positive 10-year AL (%)*	*χ* ^*2*^	*df*	*p*
District = Wakiso	69.6	4.43	1	.035*
District = Mukono	50.9			
Volunteering to clean the health facility = yes	85.7	8.87	1	.003*
Volunteering to clean the health facility = no	52.0			

*Statistically significant based on a 95% confidence interval

**Table 4:** Bivariate analysis of associations between VHT factors and 10-year anticipated longevity: continuous and scale^a^ variables

*Continuous variable*	*Mean difference* ^*b*^ *positive - negative 10-year AL*	*Standard error difference*	*t*	*df*	*p*
Years working as a VHT	0.451 years	0.335	1.34	123	.181
*Rating scale* ^*a*^ *variable*					
Greater ease of home visiting	0.641	0.210	3.05	128	.003*
Less challenge with transportation	0.368	0.194	1.90	129	.059
Less challenge with lack of supervision	0.121	0.259	0.47	123	.641
Less challenge with lack of appreciation/respect	0.385	0.270	1.43	129	.157
Less challenge with insufficient supplies	−0.209	0.178	−1.23	129	.243
Less challenge with lack of financial support	0.247	0.211	1.7	129	.245
Less challenge with lack of skills or knowledge	0.104	0.281	0.7	126	.713
Better quality support from family and friends	0.522	0.253	2.06	128	.041
Better quality support from local community	0.538	0.233	2.31	129	.022*
Better quality support from local health center	0.741	0.258	2.87	128	.005*
Better quality support from government health offices	0.682	0.243	2.81	127	.006*
Greater level of supervision	0.447	0.247	1.81	128	.073
Greater level of VHT meetings	0.211	0.234	0.90	129	.369
Greater level of respect and appreciation	0.840	0.248	3.39	125	.001*
Greater level of learning useful skills	0.531	0.260	2.04	128	.043*
Greater level of supplies to distribute	0.159	0.214	0.75	128	.457
Greater level of transportation tools (bicycles, boots)	0.348	0.188	1.85	127	.067
Greater level of partnership with health center staff	0.770	0.223	3.5	125	.001*

^a^Scale variables were on a 1–5 rating scale

^b^Difference of mean continuous value or rating score between those reporting positive 10-year AL and those reporting negative 10-year AL

*Statistically significant based on a 95% confidence interval

VHT subjects in the Wakiso District had a significantly higher proportion of positive 10-year anticipated longevity (*χ* ^*2*^ = 4.43, *df* = 1, *p* < .05). Consequently, the bivariate analysis was stratified by district, which revealed confounding in the initially significant associations between anticipated longevity and support from the local community, support from government health offices, and learning useful skills. When stratified, there remain significantly positive associations between 10-year anticipated longevity and greater ease with home visiting (Wakiso *t* = 2.06, *df* = 42; Mukono *t* = 2.21, *df* = 84; *p* < .05), greater level of partnership with health center staff (Wakiso *t* = 2.40, *df* = 19; Mukono *t* = 2.09, *df* = 82; *p* < .05), and volunteering to clean the health facility (Wakiso *χ* ^*2*^ = 4.0, *df* = 1; Mukono *χ* ^*2*^ = 5.66, *df* = 1; *p* < .05). In Mukono, positive 10-year anticipated longevity has a significantly positive association with greater levels of social support through respect and recognition (*t* = 2.65, *df* = 80, *p* < .05) and better quality support from family and friends (*t* = 2.03, *df* = 83, *p* < .05) and the health center (*t* = 2.35, *df* = 83, *p* < .05), but these associations were not significant in Wakiso. These social supports were, in fact, received at greater levels in Wakiso compared to Mukono.

### Descriptive results and thematic analysis of challenges for individual VHTs

In categorical responses, more than 75% of VHTs rated insufficient supplies (e.g. medicines to distribute), transportation difficulties, and lack of financial support as “challenging” or “very challenging.” In free responses, the most commonly discussed challenge was lack of specific material supplies, notably rubber boots (“gumboots”) and medications to distribute. The other commonly discussed challenge involved resistance from the community and lack of respect or role legitimization as health workers. Others reported being belittled by community members and other health workers.

- “We find it hard to monitor latrines, especially in the rainy season when we don't have gumboots. We use bare feet.” (Participant 095, VHT member)
- “When we go to visit households…[they] tell us to leave them alone because they are working for money but we are only volunteers.” (Participant 107, VHT member)
- “...people we home visit minimize us.” (Participant 001, VHT member)
- “[Health center staff] take us to be people who are not educated.” (Participant 102, VHT member)

The problem of inconsistent provision of supplies and other support was also noted by several key informants as potentially causing loss of morale and lack of legitimacy as a health worker for VHTs. Carrying a supply of medicines to distribute is commonly understood throughout Uganda as a defining characteristic of the *omusawo*, the health worker. Without such supplies, the very legitimacy of VHTs as true health workers may be called into question. Additionally, key informants noted how the difficulties VHTs face are compounded by the common struggle to make a livelihood in addition to volunteer health work.

- “People are working on daily bread.” (Participant 138, District health official)
- “...[M]aking a living is a whole time affair.” (Participant 136, Ugandan government official)

### Thematic analysis of challenges and opportunities for the VHT program at the system level

Informants viewed the VHT program as making a positive impact in Uganda’s health system and saw VHTs as both underutilized and under-supported. Some reported that the national government lacks a political commitment to the VHT program and that local governments lack capacity to manage the VHT program and integrate it into the broader health system. The VHT program has become dependent on NGO implementing partners (IPs), which are seen largely as poorly harmonized and misaligned with domestic policy. IPs are ultimately beholden to their donor priorities and often have temporary, unpredictable commitment to the VHT program.

- “People come with their programs and they do what they do and they quit.” (Participant 135, NGO program manager).

Some informants view regularly providing VHTs with government-funded material or monetary support as financially unfeasible and unsustainable.

- “[Implementing partners] are very welcome to do what they can, because we would be doing that ourselves, as a government, as a country... But we can’t do it because we cannot afford it.” (Participant 136, Ugandan government official)

Informants discussed the need for greater local and national ownership of the VHT program exercised through stronger governance authority and ownership. They discussed the need for IPs to operate in a more harmonious way across the country, aligning their agendas and operations with MOH policies, and for the VHT program to be integrated into broader health and social systems.

- “Districts have to have enough muscle…to own the VHT programs, to direct [implementing partners] and people who come in to support.” (Participant 135, NGO program manager)

## Discussion

The results of this study illuminate a number of key support needs for VHTs, challenges they face, and factors associated with long-term retention. VHTs need a reliable supply of basic materials, such as medicines to distribute and rubber boots, as well as transportation allowances. VHTs commonly face difficult working conditions and threats to dignity that may accelerate attrition. Retention would be increased through efforts to promote stronger partnerships between VHTs and other health workers and provide the aforementioned essential supplies and allowances. These results align with the recent MOH assessment, indicating that, while viable, Uganda’s VHT program is in need of strengthening in a number of key areas [4].

### VHT support needs

We found that VHTs prioritize receiving additional support in the forms of rubber boots (“gumboots”), bicycles, umbrellas, medicines to distribute, and uniforms. These materials would make standard tasks such as home visiting easier and may also enhance VHT status in the community. Monthly remuneration would potentially increase long-term retention among 27% of respondents. The most commonly desired form of remuneration is a transportation allowance. Ministry of Health guidelines state that local governments should devise “innovative financing mechanisms” to provide VHTs with a monthly allowance of UGX 10,000 [5]. It does not appear that such mechanisms are reliably or uniformly present in the study area. A modest and consistently provided transportation allowance of UGX 10,000 per month (USD 3.0) [22] would likely improve job satisfaction and potentially increase retention among VHTs. Given the size of the VHT program, the feasibility and sustainability of financial compensation for VHTs should be examined with consideration of the cost of turnover and the benefits of retention [29–31].

### VHT challenges at the individual and system levels

We found that there is substantial variability and inconsistency in the source, distribution, amount, quality, and sustainability of the support provided to VHTs. In particular, VHTs reported consistently low provision of transportation tools such as gumboots or bicycles. While the majority of VHTs in this study are highly motivated by a desire to improve health, many survive by subsistence farming or fishing. According to our findings and other literature, VHT work commonly involves walking long distances over poorly maintained roads, inspecting fetid latrines, and receiving requests for medicines but often having none to give [4]. A VHT member’s social status as an *omusawo*, a health worker, may deteriorate if she has no medicines to provide. Although their volunteer work costs valuable time [18] and involves difficult conditions, nearly half of VHTs report receiving little or no respect or appreciation in return. Some VHTs described being disparaged or resisted by professional health workers and members of the community. Our data and other reports indicate that this undignified treatment is due, at least in part, to being perceived as having low levels of education and volunteer status [4]. Thus, our findings indicate that the dignity of the VHT member may often be under threat.

At the system level, there appears to be limited political inclination or resources to increase domestic funding for the large VHT workforce, which is approaching 200,000 members [4]. Government funding for the health sector has fallen short of established targets [32] and spending shortfalls for the health workforce are well known [33, 34]. Thus, the VHT program remains reliant on implementing partners (IPs), which our sources describe as tending towards brief timelines and poor harmonization and alignment [35]. These system level challenges raise the question of who is truly accountable for the program’s success or failure.

### VHT retention

We used 10-year anticipated longevity (AL) as a proxy estimate of long-term retention. We found that retention may likely be greater when VHTs have supportive, productive relationships with professional health workers at local health centers. This is an essential aspect of health systems strengthening and has been identified elsewhere as a key source of health worker motivation and retention [21, 36, 37]. Further study is needed on innovative ways to catalyze these health worker partnerships in Uganda, building on existing models [38]. Retention is also likely greater when standard VHT tasks such as home visiting are made easier. This could be pursued both by providing needed materials such as gumboots and transportation allowances and by elevating VHT status in the community, for example, by building stronger partnerships between VHTs and professional health workers at the local health center. Finally, our finding that volunteering to clean the health center is positively associated with 10-year AL may reflect a deep commitment and sense of engagement in the healthcare team among a portion of VHTs.

Although many VHTs live difficult lives and face threats to their dignity, they appreciate the purpose and value of their work and are motivated to improve health and serve their communities. Sustainable measures to build partnerships between health workers and provide essential supplies and allowances have the potential to increase retention, promote dignity, and increase the health impact of VHTs. This is a “people-centered approach” to the health system, that is, purposefully engaging each individual’s perspective and agency [39].

### Key differences between the study areas

Although both areas had commonalities, VHTs in the Wakiso district had a higher proportion of 10-year AL and greater social support such as respect and support from family and the health center compared to those in Mukono. We did not explore these differences in depth, but an important factor to note is that of IP involvement. With support from UNICEF, Malaria Consortium, and, most recently, the Global Fund’s “new funding model,” a robust integrated Community Case Management (iCCM) program has been implemented in Wakiso, whereby VHTs receive ongoing training, supportive supervision, and medicines to distribute [40]. At the time this data was collected, Mukono had not yet seen the same level of resource inputs for VHTs. Based on our findings, inputs such as the Global Fund's iCCM model may prove to be a powerful tool to support VHTs if these can be equitably implemented and sustained, which is a well-known challenge [41, 42]. Further study is needed to assess how diagonal funding streams such as the Global Fund’s support of iCCM can be sustained to effectively support and retain VHTs [40, 43].

### Limitations

There is significant cultural, geographical, and socioeconomic diversity throughout Uganda. Although our findings largely align with the Ministry of Health’s national VHT assessment, the generalizability of these findings is limited due to the sample size and selection from only two districts in central Uganda. The predictions of 10-year anticipated longevity are self-reported, rough estimates of retention and surrogates for actual retention rates. Surveys and interviews run the risk of response bias based on what is socially desirable or a desire to acquiesce [44]. Although some extent of social bias is unavoidable, we addressed this limitation by ensuring all Likert item rating scales were identically balanced, ensuring that data collectors were not known to VHT respondents, and regularly reminding respondents of the anonymity and confidentiality of their responses.

## Conclusions

Uganda’s VHTs promote health equity by delivering essential interventions to people whose health care access is otherwise limited. This study highlighted the support needs of VHTs and showed how individual and system-level challenges impact their everyday work. We used VHTs’ 10-year anticipated longevity to estimate long-term community health workforce retention—a difficult aspect of health system functioning to measure. Our findings align with other evidence, suggesting that building stronger partnerships between VHTs and other health workers is an important means of supporting and retaining VHTs. External funding streams, specifically for providing supplies and allowances, can enhance VHT programs and improve retention, but the question of sustainability remains. We hope these findings inform people-centered efforts to support and retain VHTs and consequently strengthen equitable community health care delivery in Uganda.

## Additional file

Additional file 1: VHT Survey. (PDF 724 kb)

## Abbreviations

AL: Anticipated Longevity
CHW: Community Health Worker
iCCM: integrated Community Case Management
IP: Implementing Partner
MOH: Ministry of Health, Uganda
NGO: Non-governmental Organization
TASO: The AIDS Support Organization
UNICEF: United Nations Children’s Emergency Fund
USD: U.S. Dollar
VHT: Village Health Team (member)
