# Supplementary material for: Supporting and retaining Village Health Teams: an assessment of a community health worker program in two Ugandan districts
Source: Int J Equity Health. 2017 Jul 20;16:129. doi: 10.1186/s12939-017-0619-6 (PMC5520299; doi:10.1186/s12939-017-0619-6)
Supplement: Additional file 1: — VHT Survey. (PDF 724 kb) [file 12939_2017_619_MOESM1_ESM.pdf]

Okunonyereza kuno kulimu ebibuuzo kubyoyisemu nga VHT member oba (omusawo owokukyalo). Ebibuuzo ebimu tukuwa ebyokusalowo ate ebirala bya kupimapima okuva ku 1 okutuka ku 5. Dembe lyo okudamu ekibuuzo oba okukileka notajuzawo. Tewali kidibwamu kituufu oba kikyamu. Kyona kyonodamu kyakukumibwa nga kyakyama nólwekyo ddamu nga oyogera mazima n’abwenkanya. Ekigendererwa kyókunonyereza kuno kwekulaba nga tutegera bulungi embeera ya ba VHT members gye bayitamu. Twagala okuyiga kiki ddala ekitufu kyolowooza.

## OKUGEZESA OKUGERAGERANYA

Ebibuzo ebisinga mu kunonyereza kuno kwagala ogerageranya ebintu. Ekyokulabirako kya kino engeri bwe tunakola: Okupima okusoka **1** (ssikyagalilako ddala) okutuka ku **5** (nkyagala nnyo) okugerageranya bino wamanga tugerageranya mmere okusinzira gyo’yagala ne gyo’tayagala

| | 1 Ssikyagalirako ddala | 2 Sikyagala | 3 Wakatikati | 4 Nkyagala | 5 Nkyagala nnyo |
|---|---|---|---|---|---|
| Matooke | ❑ | ❑ | ❑ | ❑ | ❑ |
| Ekyenyanja | ❑ | ❑ | ❑ | ❑ | ❑ |
| Enanansi | ❑ | ❑ | ❑ | ❑ | ❑ |
| Chokuleti | ❑ | ❑ | ❑ | ❑ | ❑ |
| Ebilara (*bambi birage*) | ❑ | ❑ | ❑ | ❑ | ❑ |

This survey contains questions about your experience as a Village Health Team (VHT) member. Some questions will ask you to select different answer choices; other questions will ask you to rate things on a scale from 1 to 5. If you prefer not to answer a question, simply leave the question blank. There are no right or wrong answers. Your answers in this questionnaire will be kept confidential, so you are free to answer honestly. The goal of this research is to better understand the experience of Village Health Team (VHT) members. We want to learn what you truly think.

## PRACTICE RATING

Several questions in this survey will ask you to rate things. Here is an example of how this works:
On a scale of **1** (I do not like it all) to **5** (I like it a lot), rate the following food options according to what you like and do not like.

| | 1<br>**I do not like it at all** | 2<br>**I do not like it** | 3<br>**In the middle** | 4<br>**I like it** | 5<br>**I like it a lot** |
|---|---|---|---|---|---|
| Matoke | ❑ | ❑ | ❑ | ❑ | ❑ |
| Fish | ❑ | ❑ | ❑ | ❑ | ❑ |
| Pineapple | ❑ | ❑ | ❑ | ❑ | ❑ |
| Chocolate | ❑ | ❑ | ❑ | ❑ | ❑ |

## EKITUNDU EKISOOKA 1 – AMAWULIRE AGASOOKERWAKO AGABULIJO



1. District ki ne omuluka mwokolera nga VHT memba?

2. Laga ekiti kyemyaka gyo mwogwa

- ❑ 18-29
- ❑ 30-39
- ❑ 40-49
- ❑ 50-59
- ❑ 60-69
- ❑ 70 n'okusingawo

3. Laga ekikulakyo

- ❑ Mukazi
- ❑ Musajja

## SECTION 1 – BASIC INFORMATION



1. What is the district and parish in which you work as a VHT member?

2. Please select your age range

- ❑ 18-29
- ❑ 30-39
- ❑ 40-49
- ❑ 50-59
- ❑ 60-69
- ❑ 70 and older

3. Please select your gender

- ❑ Female
- ❑ Male

4. Mulimu ki omulala gwókola nga olese ogwa VHT, olinayo, obuvunanyizibwa obulala bwona? Laga nga wetolooza namba, okulaga omulimu o'gwo wamanga.

- ❑ Nina bizinesi oba ekibiina (okugeza eduuka omudaala)
- ❑ Mukoziwa gavument (okugeza osomesa, police, ne birala)
- ❑ Ndi muvuzi (okugeza bodaboda, takisi, ne birala)
- ❑ Nkola mu byókutunda (okugeza okukola musitoowa)
- ❑ Ndi mulimi oba muvubi okusobola okufuna emmere eyokutunda
- ❑ Niima oba nvuba okusobola okufuna kye tulya na bamaka gange
- ❑ Okolagana na bya'mikono (okugeza obubazi, okuzima, ne birala)
- ❑ Nkola mu office (okugeza nga omuwandiisi, ne birala)
- ❑ Nkola mu kolero (okugeza vanila, mwanyi, kasoli, ne birala)
- ❑ Nkola ku ddwaliro (okugeza omuzalisa, okugaba eddagala, ne birala)
- ❑ Okulabirira abaana
- ❑ Nkola ku kompuuta
- ❑ Nebirara

*Bambi birage*:

4. In addition to your work as a VHT member, what are your other occupations and daily responsibilities? Select all that apply.

- ❑ Own or operate a business or organization
- ❑ Civil Servant (for example, teacher, police, etc.)
- ❑ Driver (for example, boda-boda, taxi, etc.)
- ❑ Sales and services (for example, working in a store)
- ❑ Agriculture or fishing in order to sell your foods
- ❑ Agriculture or fishing in order to feed yourself and your family
- ❑ Skilled trade (for example, carpentry, construction work, etc.)
- ❑ Clerical work (for example, secretary in an office)
- ❑ Factory work (for example, vanilla, coffee, kasoli, etc.)
- ❑ Health care work (for example, midwife, nurse)
- ❑ Caring for children
- ❑ Technology or computer work
- ❑ Other

*Please specify*:

## EKITUNDU EKYOKUBIRI 2 – EBYAFAYO BYA VHT



5. Kiki ekyakusikiliza okubeera omunakyewa VHT? Wamanga waliwo ensonga ezimu lwaki abantu babeera ban'akyewa. Gerageranya nga okozesa ebyo ebikiweredwa okuva 1 (sikyamugaso) okutuuka ku 5 (kyamugaso nnyo) tusobole okumanya ekyakusikiriza nga nakye okufuuka VHT.

| | 1 Sikyamugaso | 2 Kigasa katono | 3 Kyamugaso*mu* | 4 Kyamugaso | 5 Kyamugaso nnyo |
|---|---|---|---|---|---|
| Okuyamba amaka gange ne mikwano | ❑ | ❑ | ❑ | ❑ | ❑ |
| Okuyamba balirwana bange ne kitundu | ❑ | ❑ | ❑ | ❑ | ❑ |
| Okufuna sente oba ebirabo ebirara | ❑ | ❑ | ❑ | ❑ | ❑ |
| Okwongera ku byóbulamu n'okutaasa obulamu | ❑ | ❑ | ❑ | ❑ | ❑ |
| Nsobole okumanyika | ❑ | ❑ | ❑ | ❑ | ❑ |
| Okunyamba nfune omulimu oba okwongera mu mulimu gwange | ❑ | ❑ | ❑ | ❑ | ❑ |
| Kubanga banonda oba bansaba okubeera nakyewa | ❑ | ❑ | ❑ | ❑ | ❑ |
| Okufuna amagezi n'obukugu | ❑ | ❑ | ❑ | ❑ | ❑ |
| Nebirala (*bambi birage*) | ❑ | ❑ | ❑ | ❑ | ❑ |

## SECTION 2 - VHT MEMBER BACKGROUND



5. What motivated you to volunteer as a VHT? Below are some reasons why people volunteer. Please rate the following options from 1 (not important) to 5 (very important) so we can learn what was important to you when you volunteered to become a VHT.

| | 1 Not important | 2 A little important | 3 In the middle | 4 Important | 5 Very important |
|---|---|---|---|---|---|
| To help my family and friends | ☐ | ☐ | ☐ | ☐ | ☐ |
| To help my neighbors and community | ☐ | ☐ | ☐ | ☐ | ☐ |
| To receive money or other reward | ☐ | ☐ | ☐ | ☐ | ☐ |
| To improve health and save lives | ☐ | ☐ | ☐ | ☐ | ☐ |
| To receive recognition | ☐ | ☐ | ☐ | ☐ | ☐ |
| To help me get a job or improve my career | ☐ | ☐ | ☐ | ☐ | ☐ |
| Because I was elected or asked to volunteer | ☐ | ☐ | ☐ | ☐ | ☐ |
| To gain useful knowledge and skills | ☐ | ☐ | ☐ | ☐ | ☐ |
| Other (please specify) | ☐ | ☐ | ☐ | ☐ | ☐ |

6. Ddi lwewatendekebwa nga VHT memba? Laga omwezi n'omwaka.

7. Olabirira amayumba ameka nga VHT memba?

8. Mussabiti emu tebereza esawa meka z'omala nga okola emirimu gy'obwa VHT?

6. When were you trained as a VHT member? Please write the month and year.

7. For how many households are you responsible as a VHT member?

8. In a typical week, what is the estimated total hours you spend performing your work as a VHT?

## EKITUNDU EKYOKUSATU 3 – EBIKOLEBWA BA VHT



9. Wali wetabyemu ku bikolebwa mu Ministry ye byo'bulamu ku District? Bambi laga ekyo kyewetabamu.

- ❑ Enaku ezokugema
- ❑ Okugaba obutimba bwensiri obulimu eddagala
- ❑ Enaku zo'bulamu mu maka (famile health days)
- ❑ Okugaba no'kubiriza mukugaba eddagala lye'biwuka
- ❑ Okugaba eddagala lya vitamini
- ❑ Nebirala

*Bambi biwandiike*:

10. Wali wetabyamu mu bikolebwa ku kyalo nga VHT byalina okukola? Bambi laga byona byo'sobodde okwetabamu.

- ❑ Okukyalira amaka
- ❑ Okwetaba mu nkiiko ezokubaganya ebilowoozo eza ba VHT
- ❑ Okutendekebwa mu ndwadde ezisobola okujanjabirwa awaka oba mu kitundu (ICCM)
- ❑ Okwetaba mu nkiiko ze kitundu n'abakulembeze mu kitundu
- ❑ Okuzimba ebyotto
- ❑ Okuzimba nokulongosa enzizi (ensibuko za mazzi)
- ❑ Nebirala

*Bambi biwandiike*:

## SECTION 3 - VHT MEMBER ACTIVITIES



9. Have you participated in Ministry of Health/District Health Office activities? Please select all that apply.

- ❑ Immunisation days
- ❑ ITN (insecticide-treated mosquito net) distribution
- ❑ Family health days
- ❑ De-worming campaigns
- ❑ Vitamin distribution
- ❑ Other

*Please specify*:

10. Have you participated in local VHT activities? Please select all that apply.

- ❑ Home visiting
- ❑ Focus groups
- ❑ ICCM training (Integrated Community Case Management of childhood diseases)
- ❑ Community meetings or meetings with community leaders
- ❑ Cookstove projects
- ❑ Protected water source projects
- ❑ Other (please specify)

*Please specify*:

11. Mu mwaka gumu emabega oguyise obade okola otya emirimu gino wamanga? Nga ogerageranya okuva ku 1 (sikolangako) okutuka ku 5 (nkola bulikasera), bambi tulage engeri gyobadde okolamu buli mulimu.

| | 1 **Sikikolangako** | 2 **Sitera kukikola** | 3 **Bwentyo-bwentyo** | 4 **Ebiseera ebimu** | 5 **Ebiseera ebisinga** |
|---|---|---|---|---|---|
| Okukyalira amaka | ☐ | ☐ | ☐ | ☐ | ☐ |
| Okuba mu nkiiko za ba VHT | ☐ | ☐ | ☐ | ☐ | ☐ |
| Okubudabuda n'okusomesa mu byóbulamu | ☐ | ☐ | ☐ | ☐ | ☐ |
| Okubiliza abantu mu kitundu oku kozesa edwaliro mu byóbulamu | ☐ | ☐ | ☐ | ☐ | ☐ |
| Okuyamba ku dwaliro erikuli okumpi | ☐ | ☐ | ☐ | ☐ | ☐ |
| Okusobola okulaba n'okujanjaba endwadde (nga lubyamira, ekiddukano, omusujja gwénsiri) mu baana | ☐ | ☐ | ☐ | ☐ | ☐ |
| Okulolondola ku bakyala abali embuto n'abaana babwe nga bawere | ☐ | ☐ | ☐ | ☐ | ☐ |
| Okulondola abantu nga babasibude okuva mu dwaliro, abali awaka nga balwadde, abalwadde bakawuka kamukenenya, ne birala | ☐ | ☐ | ☐ | ☐ | ☐ |
| Okugaba ebintu byóbulamu (nga obutimba bwénsiri) | ☐ | ☐ | ☐ | ☐ | ☐ |
| Okulaba obubonero bwóbulwadde mu maka | ☐ | ☐ | ☐ | ☐ | ☐ |
| Okuwandiika ebyo byokola n'okuwayo alipota | ☐ | ☐ | ☐ | ☐ | ☐ |
| Okusisinkanako munkiiko za ba VHT bano | ☐ | ☐ | ☐ | ☐ | ☐ |
| Okwetaba mu mirimu egikolebwa mu byóbulamu ku kitundu (okugeza nga okuzimba ebyoto) | ☐ | ☐ | ☐ | ☐ | ☐ |
| Ebilara (*bambi birage*) | ☐ | ☐ | ☐ | ☐ | ☐ |

11. Over the last year, how often have you performed each of the following activities? On a scale from 1 (never) to 5 (often) please rate how frequently you have performed each activity.



| | 1 Never | 2 Rarely | 3 In the middle | 4 Sometimes | 5 Often |
|---|---|---|---|---|---|
| Home visiting | ❑ | ❑ | ❑ | ❑ | ❑ |
| VHT meetings | ❑ | ❑ | ❑ | ❑ | ❑ |
| Health counseling and education | ❑ | ❑ | ❑ | ❑ | ❑ |
| Mobilizing your community to utilize health services | ❑ | ❑ | ❑ | ❑ | ❑ |
| Helping at the local health centre | ❑ | ❑ | ❑ | ❑ | ❑ |
| Identifying and managing childhood illness (pneumonia, diarrhea, malaria) | ❑ | ❑ | ❑ | ❑ | ❑ |
| Following up on pregnant mothers and mothers with newborns | ❑ | ❑ | ❑ | ❑ | ❑ |
| Following up on people who have been discharged from health facility, those who are sick at home, HIV+ people, etc. | ❑ | ❑ | ❑ | ❑ | ❑ |
| Distributing health commodities (for example, mosquito nets) | ❑ | ❑ | ❑ | ❑ | ❑ |
| Monitoring for signs of illness in households | ❑ | ❑ | ❑ | ❑ | ❑ |
| Record keeping and submitting reports | ❑ | ❑ | ❑ | ❑ | ❑ |
| Meeting with other local VHT members | ❑ | ❑ | ❑ | ❑ | ❑ |
| Other local health projects (for example, cookstoves) | ❑ | ❑ | ❑ | ❑ | ❑ |
| Other *(please specify)* | ❑ | ❑ | ❑ | ❑ | ❑ |

## EKITUNDU EKYOKUNA 4 – BYOYISEMU MU KUBEERA VHT MEMBA



12. Wansi gye mirimu egilina okukolebwa VHT memba nga ministule ye byóbulamu bweyategeka. Nga okozesa okupima okuva ku 1 (kizibu nnyo) okutuka ku 5 (kyangu nnyo) gerageranya obuzibu n′obwangu bwobadde nabwo nga okola buli mulimu guno.

| | 1 **Kizibu nnyo** | 2 **Kizibuzibu** | 3 **Kizibu*mu*** | 4 **Kyangungu** | 5 **Kyangu nnyo** |
|---|---|---|---|---|---|
| Okukyalira amaka | ❑ | ❑ | ❑ | ❑ | ❑ |
| Okubiriza abantu mu kitundu okukozesa edwaliro | ❑ | ❑ | ❑ | ❑ | ❑ |
| Okutumbula ebyóbulamu n′okusomesa | ❑ | ❑ | ❑ | ❑ | ❑ |
| Okusobola okulabirira abantu mu ndwadde enyangungu (ICCM) | ❑ | ❑ | ❑ | ❑ | ❑ |
| Okulondora abakyala abali embuto wamu n′abaana abawere wamu nokubawereza kuddwaliro nga balaze obubonero obwakabi | ❑ | ❑ | ❑ | ❑ | ❑ |
| Okulondora abantu ababa basiibudwa oluvanyuma lwókulwara | ❑ | ❑ | ❑ | ❑ | ❑ |
| Okulondora abantu abali kubujanjabi obwekissera ekiwanvu (okugeza nga abakafuba oba mukenenya) | ❑ | ❑ | ❑ | ❑ | ❑ |
| Okugaba ebintu byóbulamu | ❑ | ❑ | ❑ | ❑ | ❑ |
| Okutereka n′okuwandiika amawulire gomu kintundu | ❑ | ❑ | ❑ | ❑ | ❑ |
| Okulaba n'okulondoola endwadde ezibalusewo mu kitundu | ❑ | ❑ | ❑ | ❑ | ❑ |

## SECTION 4 - EXPERIENCE BEING A VHT MEMBER



12. Below are the duties of every VHT member as established by the Ministry of Health. On a scale from 1 (very difficult) to 5 (very easy) rate the difficulty or ease you have performing each activity.

| | 1 Very difficult | 2 Somewhat difficult | 3 In the middle | 4 Somewhat easy | 5 Very easy |
|---|---|---|---|---|---|
| Home visiting | ❑ | ❑ | ❑ | ❑ | ❑ |
| Mobilizing the community to utilize health services | ❑ | ❑ | ❑ | ❑ | ❑ |
| Health promotion and education | ❑ | ❑ | ❑ | ❑ | ❑ |
| Managing common illness conditions (ICCM) | ❑ | ❑ | ❑ | ❑ | ❑ |
| Following up on pregnant mothers and newborns and referring when there are danger signs | ❑ | ❑ | ❑ | ❑ | ❑ |
| Following up on people who were discharged from the health centre after an illness | ❑ | ❑ | ❑ | ❑ | ❑ |
| Following up on people who are taking long-term treatment (for example TB or HIV treatment) | ❑ | ❑ | ❑ | ❑ | ❑ |
| Distributing health commodities | ❑ | ❑ | ❑ | ❑ | ❑ |
| Record keeping of community information | ❑ | ❑ | ❑ | ❑ | ❑ |
| Monitoring for diseases in the community | ❑ | ❑ | ❑ | ❑ | ❑ |

13. Kusomozebwa ki oba bizibu ki byolina nga VHT memba? Bangi ba VHT basanga ebizibu ebibaremesa okukola emirimu gyabwe nga ba VHT. Piima okuva ku 1 (egamba nti kisomozo nnyo) okutuka ku 5 (egamba nti sikisomozo nakamu) wamanga bye bizibu oba bisomoza byoyisemu.



| | 1 **Kisomozo nnyo** | 2 **Kisomozo** | 3 **Kisomoza*mu*** | 4 **Kisomoza katono** | 5 **Sikisomozo nakamu** |
|---|---|---|---|---|---|
| Entambula | ❑ | ❑ | ❑ | ❑ | ❑ |
| Obutakubirizibwa | ❑ | ❑ | ❑ | ❑ | ❑ |
| Obutawebwa kitiibwa n'okusiimibwa okuva mu bantu mukitundu ne mu malwaliro | ❑ | ❑ | ❑ | ❑ | ❑ |
| Sifuna bikozesebwa bimala | ❑ | ❑ | ❑ | ❑ | ❑ |
| Obutayambibwa mu by'esente oba okusasurwa | ❑ | ❑ | ❑ | ❑ | ❑ |
| Obutaba na bumanyirivu oba amagezi | ❑ | ❑ | ❑ | ❑ | ❑ |

13a. Waliyo ebizibu ebirara byoyitamu nga okola omulimu gwo nga VHT? Bwekiba nti gyebiri, bambi binyonyore.

14. Osobode okuvunuka ebimu kubizibu ebyo ebyogedwako waggulu? Bwoba osobode, bambi tubulire ngeri gye wabivunukamu.

13. What are your challenges working as a VHT member? Many VHTs encounter challenges that limit their ability to do their work. On a scale from 1 (very challenging) to 5 (not challenging at all) rate the following challenges from your experience.

| | 1 Very challenging | 2 Challenging | 3 In the middle | 4 A little challenging | 5 Not at all challenging |
|---|---|---|---|---|---|
| Transportation | ☐ | ☐ | ☐ | ☐ | ☐ |
| Lack of supervision | ☐ | ☐ | ☐ | ☐ | ☐ |
| Lack of appreciation or respect from the community and the health centre | ☐ | ☐ | ☐ | ☐ | ☐ |
| Not enough supplies | ☐ | ☐ | ☐ | ☐ | ☐ |
| Lack of financial support or payment | ☐ | ☐ | ☐ | ☐ | ☐ |
| Lack of skills or knowledge | ☐ | ☐ | ☐ | ☐ | ☐ |

13a. Are there other challenges you experience in your work as a VHT member? If so, please describe them.

14. Have you been able to overcome any of those challenges mentioned in the previous questions? If so, please describe how.

## EKITUNDU EKYOKUTANO 5 – OBUYAMBI BWA BA VHT MEMBA



Ebibuuzo ebidako bikubuuza engeri gyoyambibwamu nga VHT memba. “Obuyambi” kitegeza ekintu kyona ekiyamba okukola obulungi omulimu nga VHT memba. Obuyambi obwokulabira ko nga okusimibwa n’okuwebwa ekitiibwa, okukola enkiiko za ba VHTs, okubiliizibwa n’okutendekebwa mu bintu ebimu ebyóbulamu.

15. Ani okuyamba mu mulimu gwo nga VHT? Geragerany a okusinzira ku kikuweredwa wamanga okuva ku 1 (tewali buyambi) okutuuka ku 5 (obuyambi obukyasinze), geragerany a okusinziira kubuyambi bwewakafuna okuva mu biibinja bino wamanga.

| | 1 Tewali buyambi | 2 Obuyambi butono | 3 Obuyambi bwa wakatikati | 4 Obuyambi bulungi | 5 Obuyambi obukyasinze |
|---|---|---|---|---|---|
| Abamaka gange n’emikwano | ☐ | ☐ | ☐ | ☐ | ☐ |
| Abantu mu kintundu kyange | ☐ | ☐ | ☐ | ☐ | ☐ |
| Edwaliro ebyómukintundu | ☐ | ☐ | ☐ | ☐ | ☐ |
| Ministry ye byóbulamu | ☐ | ☐ | ☐ | ☐ | ☐ |
| Ebiibina ebitali bya gavumenti ebikwatagana ne VHTs (*bambi birage*) | ☐ | ☐ | ☐ | ☐ | ☐ |
| Ebilara (*bambi birage*) | ☐ | ☐ | ☐ | ☐ | ☐ |

## SECTION 5 - SUPPORT FOR THE VHT MEMBER



The following questions will ask how you are supported as a VHT member. "Support" means anything that helps you work well as a VHT member. Examples of support you may have received in your work include things like appreciation and respect, VHT meetings, supervision, and ongoing training.

15. Who supports you in your work as a VHT? On a scale from 1 (no support) to 5 (the best support), please rate the support you currently receive from the following groups.

| | 1 No support | 2 A little support | 3 In the middle | 4 Good support | 5 The best support |
|---|---|---|---|---|---|
| My family and friends | ❑ | ❑ | ❑ | ❑ | ❑ |
| My local community | ❑ | ❑ | ❑ | ❑ | ❑ |
| The local health centre | ❑ | ❑ | ❑ | ❑ | ❑ |
| The District Health Office/Ministry of Health | ❑ | ❑ | ❑ | ❑ | ❑ |
| A non-government VHT organization (please specify) | ❑ | ❑ | ❑ | ❑ | ❑ |
| Other (please specify) | ❑ | ❑ | ❑ | ❑ | ❑ |

16. Ngeri ki ez'omugaso ezisobola okuyamba n'okuzamu amanyi mu kukola emirimu gya VHT? Wansi waliwo engeri VHT gyayinza okuyambibwa n'okuzibwamu amanyi. Okuva ku kyokudamu 1 (sikyamugaso) okutuuka ku 5 (kyamugaso nnyo), gerageranya kwebyo olondeko.



| | 1 **Sikyamugaso** | 2 **Kigasa katono** | 3 **Kyamugaso*mu*** | 4 **Kyamugaso** | 5 **Kyamugaso nnyo** |
|---|---|---|---|---|---|
| Okubirizibwa | ❑ | ❑ | ❑ | ❑ | ❑ |
| Enkiiko ne ba VHTs abalala | ❑ | ❑ | ❑ | ❑ | ❑ |
| Okufuna ekitiibwa n'okusimibwa | ❑ | ❑ | ❑ | ❑ | ❑ |
| Okusomesebwa obukodyo obwomugaso mu byóbulamu | ❑ | ❑ | ❑ | ❑ | ❑ |
| Ebintu ebyókugaba nga edagala eryebiwuka n'obutimba bwe nsiri | ❑ | ❑ | ❑ | ❑ | ❑ |
| Okutendekebwa obukodyo obupya mu ba VHTs | ❑ | ❑ | ❑ | ❑ | ❑ |
| Ebiyamba muntambula nga eggali oba butusi | ❑ | ❑ | ❑ | ❑ | ❑ |
| Enkolagana na basawo kudwaliro elyómukitundu kyo | ❑ | ❑ | ❑ | ❑ | ❑ |
| Ebilara (*bambi birage*) | ❑ | ❑ | ❑ | ❑ | ❑ |

16. What are the important ways that you can be motivated and supported to perform your work as a VHT? Below are some ways VHTs can be supported and motivated. On a scale from 1 (not important) to 5 (very important), please rate the following options.

| | 1 **Not important** | 2 **A little important** | 3 **In the middle** | 4 **Important** | 5 **Very important** |
|---|---|---|---|---|---|
| Supervision | ❑ | ❑ | ❑ | ❑ | ❑ |
| Meeting with other VHTs | ❑ | ❑ | ❑ | ❑ | ❑ |
| Receiving respect and appreciation | ❑ | ❑ | ❑ | ❑ | ❑ |
| Learning useful skills | ❑ | ❑ | ❑ | ❑ | ❑ |
| Supplies to distribute, such as deworming tablets or mosquito nets | ❑ | ❑ | ❑ | ❑ | ❑ |
| Training on new VHT skills | ❑ | ❑ | ❑ | ❑ | ❑ |
| Transportation tools, such as bicycles or boots | ❑ | ❑ | ❑ | ❑ | ❑ |
| Partnership with staff at the local health centre | ❑ | ❑ | ❑ | ❑ | ❑ |
| Other (*please specify*) | ❑ | ❑ | ❑ | ❑ | ❑ |

17. Buyambi bwenkana wa bwewafuna omwaka oguwedde? Wansi yengeri ezifanagana ba VHTs mwebayambibwa okuva kukyokudamu 1 (tewali nakamu) okutuka 5 (bumala) kati awono gerageranya ekyokulondako nga wesigama kwebyo olagge obuyambi bwewakafuna omwaka oguwedde.

| | 1 Tewali nakamu | 2 Butono | 3 Bwawakati awo | 4 Kalekaleko | 5 Bumala |
|---|---|---|---|---|---|
| Okubirizibwa | ☐ | ☐ | ☐ | ☐ | ☐ |
| Enkiiko ne ba VHTs abalala | ☐ | ☐ | ☐ | ☐ | ☐ |
| Okufuna ekitiibwa n’okusimibwa | ☐ | ☐ | ☐ | ☐ | ☐ |
| Okusomesebwa obukodyo obwomugaso mu byóbulamu | ☐ | ☐ | ☐ | ☐ | ☐ |
| Ebintu ebyókugaba nga edagala eryebiwuka n’obutimba bwe nsiri | ☐ | ☐ | ☐ | ☐ | ☐ |
| Okutendekebwa obukodyo obupya mu ba VHTs | ☐ | ☐ | ☐ | ☐ | ☐ |
| Ebiyamba muntambula nga eggali oba butusi | ☐ | ☐ | ☐ | ☐ | ☐ |
| Enkolagana na basawo kudwaliro elyómukitundu kyo | ☐ | ☐ | ☐ | ☐ | ☐ |
| Ebilara (*bambi birage*) | ☐ | ☐ | ☐ | ☐ | ☐ |

17. How much support have you received in the last year? Below is the same list of ways VHTs can be supported and motivated. On a scale from 1 (none) to 5 (enough), please rate the following options based on how much support you have received in the last year.

| | 1 None | 2 A little | 3 In the middle | 4 Almost enough | 5 Enough |
|---|---|---|---|---|---|
| Supervision | ❑ | ❑ | ❑ | ❑ | ❑ |
| Meeting with other VHTs | ❑ | ❑ | ❑ | ❑ | ❑ |
| Receiving respect and appreciation | ❑ | ❑ | ❑ | ❑ | ❑ |
| Learning useful skills | ❑ | ❑ | ❑ | ❑ | ❑ |
| Supplies to distribute, such as deworming tablets or mosquito nets | ❑ | ❑ | ❑ | ❑ | ❑ |
| Training on new VHT skills | ❑ | ❑ | ❑ | ❑ | ❑ |
| Transportation tools, such as bicycles or boots | ❑ | ❑ | ❑ | ❑ | ❑ |
| Partnership with staff at the local health centre | ❑ | ❑ | ❑ | ❑ | ❑ |
| Other (*please specify*) | ❑ | ❑ | ❑ | ❑ | ❑ |

18. Buyambi ki bwóyongerako obuyinza okuyamba okukola obulungi ennyo emirimu gwo?

19. Senga obuyambi bwewafuna busigala nga bwe bumu, onaba osobola okusigala nga okukola nga VHT okumala emyaka emirala 10? Wano londako kimu.

- ❑ Yee
- ❑ Neda
- ❑ Simanyi

18. What additional means of support would help you better perform your work?



19. If the support you currently receive stays the same, will you be able to continue working as a VHT member for at least 10 more years? Please select one.

- ❑ Yes
- ❑ No
- ❑ I don't know

*Bwoba wazemu "Neda" oba "Simanyi" ku kibuuzo ekyo waggulu weyongero odemu ebibuuzo 20 ne 21.*
*Bwoba wazemu "Yee" ku kibuuzo ekyo waggulu, ebibuuzo 20 ne 21 tobidamu weyongereyo ku Kitundu Ekyómukaaga 6.*

20. Mukuteberezakwo osubira onomala banga ki nga okyasobola okukola emirimu gya VHT? Londako kimu.

- ❑ Wakati we myaka 5 ne 10
- ❑ Wakati wo mwaka 1 ne 5
- ❑ Obutasuka mwaka
- ❑ Simanyi

21. Ekitongole kyebyóbulamu tebalina mbalirira oba ntegeka yonna yakusasula oba okuwa ba VHTs ebintu ebibayamba. Naye ate olwekigendererwa ekyokunonyereza kuno, byanfuna ki ***ebimala*** byoyagala ebiyinza okusigaza nga okola emyaka nga 10 mumasso?

- ❑ Sente UGX ________________ buli mwezi (*bambi birage*)
- ❑ Ssi sobola okweyongerayo kukola nga VHT mu banga mpanvu okujjako nga waliwo obuyambi obuyongedwako (*<u>bambi birage</u>)
- ❑ Ssi sente naye kintu kirara (*<u>bambi birage</u>)

*<u>Bambi birage:</u>*

*If you answered "No" or "I do not know" to the previous question, please answer questions 20 and 21.*
*If you answered "Yes" to the previous question, please skip questions 20 and 21 and proceed to Section 6.*

20. How long do you predict being able to work as a VHT member? Please select one.

- ❑ 5 to 10 more years
- ❑ 1 to 5 more years
- ❑ Less than 1 year
- ❑ I do not know

21. The Ministry of Health currently has no budget or plans to pay VHTs or provide material support. However, for the purposes of this research, what is the ***minimum*** amount of financing that would adequately support you and keep you engaged in your work for at least 10 more years?

- ❑ UGX _________________ per month (*please specify*)
- ❑ I cannot continue working as a VHT member for the long-term, regardless of additional support (*<u>please specify</u>)
- ❑ Not money, but something else (*<u>please specify</u>)

*<u>Please specify:</u>*

## EKITUNDU EKYOMUKAGA 6 – VHT MEMBA NE DDWALIRO LYÓKUKITUNDU



22. Owereza abalwadde ku ddwaliro elyókukitundu? Bwoba nga owereza, bambi wandiika onwwendo bameka botebereza okuba nti wabawereza mu myezi omukaaga egiyise.

- ❑ Yee, _____________ abantu mu myezi omukaaga egiyise
- ❑ Neda

23. Okola obwanakyewa ku ddwaliro elyókukitundu? Bwoba nga okola, wandiika esaawa zomala nga okolayo mu mwezi.

- ❑ Yee, _______ esaawa buli mwezi
- ❑ Neda

*Bwoba ekibuuzo ekyo wagulu wazemu nti yee, weyongereyo odemu ebibuuzo 24 – 29.*
*Bwoba wazemu neda, okunonyereza kati kuba kukomye.*

24. Bwokola obwanakyewa ku ddwaliro lyókukitundu, mirimu ki gyokola? Laga nga wetoloza namba yómulimu gwokolayo.

- ❑ Okupima abalwadde okugeza obuwanvu, obuzito ne buggumu, nebirala
- ❑ Okuyamba mukuwandiisa abalwadde
- ❑ Okusomesa n’okubulirira abalwadde
- ❑ Okuyamba okulongosa ku ddwaliro
- ❑ Okuyamba okuwa abalwade edagala
- ❑ Tewali mulimu gwamanyi gwenkola
- ❑ Nebirara

*Bambi birage*:

## SECTION 6 - VHT MEMBERS AND LOCAL HEALTH CENTRES



22. Do you refer patients to a local health centre? If so, please write approximately how many people you have referred in the last 6 months.

- ❑ Yes, _____________ people in the last 6 months
- ❑ No

23. Do you volunteer at a local health centre? If so, please write how many hours each month.

- ❑ Yes, _______ hours each month
- ❑ No

*If you answered yes to the previous question, please continue and answer questions 24 - 29.*
*If you answered no, the survey is now complete.*

24. When you volunteer at the local health centre, what kinds of work do you do? Select all that apply.

- ❑ Taking measurements, such as height, weight, temperature, etc.
- ❑ Helping register patients
- ❑ Teaching and counseling patients
- ❑ Helping to clean the health centre facility
- ❑ Helping in the pharmacy
- ❑ No specific tasks
- ❑ Other

*Please specify*:

25. Bwewakola obwanakyewa kudwaliro, abakozi bo kuddwaliro bakwaniriza nókuyamba? Enkolagana yo nabo yali egasa oba tegasa? Enkolagana yo yali ekuyigirizamu? Wansi ye lukalala lwe bifo byabakozi bókudwaliro. Kati lagga ekifo kyomukozi gwe wakola naye nga ogerageranya okuva ku 1 (tegasa, teyambiradala, era tebayaniriza) okutuka ku 5 (yagasa, yanyamba nnyo era eyigiriza).

| | 1 **Tebagasa, tebayambiradala, era tebayaniriza** | 2 **Bagasamu ko era bayambamu ko** | 3 **Ekiri mu makati** | 4 **Bagasamu bayambamu** | 5 **Bayambira dala nnyo era bagasiza dala ate bayigiriza** |
|---|---|---|---|---|---|
| Omusawo | ❑ | ❑ | ❑ | ❑ | ❑ |
| Omujanjabi | ❑ | ❑ | ❑ | ❑ | ❑ |
| Abakebela omusayi ne birala | ❑ | ❑ | ❑ | ❑ | ❑ |
| Ayamba ku basawo kuddwaliro | ❑ | ❑ | ❑ | ❑ | ❑ |
| Nebirala (*bambi birage*) | ❑ | ❑ | ❑ | ❑ | ❑ |

25. When you volunteer at the health centre, are the health centre staff welcoming and supportive? Are your interactions with them positive or negative? Are your interactions educational? Below is a list of health centre staff positions. Please rate the staff members you have worked with according to the support they give you on a scale from 1 (negative, unsupportive, and unwelcoming) to 5 (positive, very supportive, and educational). 

| | 1 **Negative: very unsupportive and unwelcoming** | 2 **Somewhat negative and unsupportive** | 3 **In the middle** | 4 **Somewhat positive and supportive** | 5 **Positive: very supportive and educational** |
|---|---|---|---|---|---|
| Doctors | ❑ | ❑ | ❑ | ❑ | ❑ |
| Nurses | ❑ | ❑ | ❑ | ❑ | ❑ |
| Clinical officers | ❑ | ❑ | ❑ | ❑ | ❑ |
| Support staff | ❑ | ❑ | ❑ | ❑ | ❑ |
| Other (*please specify*) | ❑ | ❑ | ❑ | ❑ | ❑ |

26. Mubyona, abasawo ku ddwaliro begatira wamu nawe nebakufula omu kubo?

- ❑ Ebiseera ebisinga
- ❑ Olusi nolusi
- ❑ Tekitera kubawo
- ❑ Tekibangawo

27. Mubyona, muli owulira nga oyambibwa abasawo ku ddwaliro?

- ❑ Yee
- ❑ Olusi nolusi
- ❑ Neda

28. Okukola obwanakyewa kuddwaliro olaba nga kikuyamba oba kiyamba gwe nga VHT?

- ❑ Yee
- ❑ Olusi nolusi
- ❑ Neda

29. Olowooza oyinza okuyambibwa obulungi abasawo kuddwaliro? Bwekiba nti yee, nyonyola engeri gyebakuyamba mu.

- ❑ Yee (*bambi nyonyola*)
- ❑ Obuyambi bwenfuna ku ddwaliro bumala.

*Bambi nynyola*:

26. In general, does the health centre staff engage with you and make you part of their team?

- ❑ Often
- ❑ Sometimes
- ❑ Rarely
- ❑ Never

27. In general, do you feel supported by the health centre staff?

- ❑ Yes
- ❑ Sometimes
- ❑ No

28. Is volunteering at the health centre a worthwhile activity for VHTs?

- ❑ Yes
- ❑ Sometimes
- ❑ No

29. Do you think you could be better supported by the local health centre and its staff? If yes, please explain how.

- ❑ Yes (please explain)
- ❑ The support I receive from the health centre is sufficient

*Please explain*:
